# Extramedullary leukemia in children presenting with proptosis

**DOI:** 10.1186/1756-8722-2-4

**Published:** 2009-01-24

**Authors:** Ramesh Murthy, Geeta K Vemuganti, Santosh G Honavar, Milind Naik, Vijayanand Reddy

**Affiliations:** 1 Ocular Oncology, Oculoplasty and Orbital Diseases, L V Prasad Eye Institute, Kallam Anji Reddy Campus, Hyderabad, India; 2 Pediatric Ophthalmology and Strabismus, L V Prasad Eye Institute, Kallam Anji Reddy Campus, Hyderabad, India; 3 Ocular Pathology, L V Prasad Eye Institute, Kallam Anji Reddy Campus, Hyderabad, India

## Abstract

**Background:**

We highlight the orbital manifestations of acute myeloid leukemia and the role of peripheral blood smear in the diagnosis of these cases. A total of 12 patients who presented with proptosis and were subsequently diagnosed to have acute myeloid leukemia based on incision biopsy or peripheral blood smear were included in the study.

**Results:**

A retrospective review of all cases of acute myeloid leukemia presenting to the Orbital clinic was performed. The age at presentation, gender, presenting features, duration of symptoms and fundus features were noted. In addition the temporal relationship of the orbital disease to the diagnosis of leukemia, laterality, location of the orbital mass, imaging features and the diagnostic tools used to diagnose leukemia were noted. The median age at presentation was 6 years. The male: female ratio was 0.7:1. None of these patients had been diagnosed earlier as having acute myeloid leukemia. The presenting features included proptosis in all patients, orbital mass in 5 (41.7%), visual symptoms in 2 (16.7%) and subconjunctival hemorrhage in one patient (8.3%). A diagnosis of acute myeloid leukemia was established by incision biopsy in 4 patients, subsequently confirmed by peripheral blood smear testing and bone marrow biopsy in 2 patients which revealed the presence of systemic involvement. Imprint smears of the biopsy identified blasts in 2 of 4 cases. In 8 patients presenting with ocular manifestations, diagnosis was established by peripheral blood smear examination alone which revealed a diagnosis of acute myeloid leukemia.

**Conclusion:**

A peripheral blood smear should be performed in all cases of sudden onset proptosis or an orbital mass in children and young adults along with an orbital biopsy. It can always be complemented with a bone marrow biopsy especially in cases of aleukemic leukemia or when the blood smear is inconclusive.

## Background

Acute myeloid leukemia (AML) accounts for nearly 15% of all leukemias in children [1]. The leukemic cells can infiltrate any extramedullary site, tumorous accumulations within soft tissues and bones being labeled as granulocytic sarcomas. Granulocytic sarcoma (GS) or extramedullary leukemic deposits is an unusual manifestation of AML, accounting for about 3% of cases of AML [2]. Allen Burns was the first to describe it in 1811 as a green tumor involving the orbit [2]. Due to their characteristic green color, King in 1853 named these tumors as chloromas (greek 'chloros' meaning green) [3]. Exposure of the myeloperoxidase enzyme present in these tumor cells to ultraviolet light is responsible for this green colour [2]. However nearly one third of the tumors do not exhibit this phenomenon. Rappaport suggested the name granulocytic sarcoma considering the association of these tumors to leukemia [4]. Granulocytic sarcoma has also been known by many other names including myeloblastoma, myelocytomas, myelosarcoma, chloroleukemia [5].

The natural history of these tumors can be variable. This tumor can present prior to, concomitantly or even during remission of systemic leukemia [5,6]. Diagnosis can be challenging if there is an orbital GS in a patient without any known hematological malignancy. Alternatively the tumor can develop in an established case of systemic leukemia. Extramedullary leukemic deposits can be seen with different forms of myeloid leukemia including acute myelogenous leukemia, chronic myelogeneous leukemia with or without blast crisis and other myeloproliferative disorders. In cases where the orbital tumor is the initial manifestation, peripheral blood and bone marrow involvement usually occurs within a year of the occurrence of orbital disease [2].

In this report we describe 12 cases of acute myeloid leukemia that presented to the ophthalmology clinic with proptosis and a diagnosis of AML was subsequently made by a peripheral blood smear or incision biopsy. This case series emphasizes the importance of these simple yet valuable investigations in making a diagnosis of this grave systemic malignancy.

## Results

Twelve cases of acute myeloid leukemia had presented with proptosis. None of these patients had been diagnosed earlier as having acute myeloid leukemia. The median age at presentation was 6 years (mean 8.6 years; range 10 months – 17 years), (Table 1). There were seven females and five males. The presenting features included proptosis in all patients, orbital mass in 5 (41.7%), visual symptoms in 2 (16.7%) and subconjunctival hemorrhage in one patient (8.3%). The median duration of symptoms before presentation was 4 weeks (mean 7 weeks; range 2 weeks to 20 weeks). Fundus manifestations in the form of retinal pale centered hemorrhages or subhyaloid hemorrhages were present in 4 (33.3%) patients and disc edema in 2 (16.7%) patients.

**Table 1:** Ocular manifestations, patient details, laterality, lymph node involvement and orbital mass location

No	Age	Sex	Presenting features	Duration of symptoms	Fundus features	Laterality	Lymph nodes involved	Exophthalmometry (mm of proptosis)	Location of mass (palpation)
1	12 years	Female	Proptosis	4 weeks	Pale centred hemorrhages, preretinal hemorrhage	Left	SMN bilateral	4 mm	Superior

2	5 years	Female	Proptosis, orbital mass	4 weeks	Normal	Bilateral	SMN, PAN, ACN bilateral	-	Superior

3	13 years	Male	Proptosis, orbital mass	8 weeks	Retinal hemorrhages	Right	PAN	9	Superotemporal

4	15 years	Male	Proptosis	4 weeks	Normal	Bilateral	Right SMN, ACN; left PAN, SMN	3	Superior

5	4 years	Female	Proptosis, orbital mass	4 weeks	Retinal hemorrhages, disc edema	Bilateral	-	5	Superotemporal

6	10 years	Female	Proptosis, orbital mass, subconjunctival hemorrhage	20 weeks	Subhyaloid hemorrhage	Left	-	8	Superotemporal

7	1 years	Female	Proptosis	2 weeks	Normal	Right	-	-	-

8	6 years	Female	Proptosis	2 weeks	Normal	Left	-	8	Superotemporal

9	4 years	Male	Proptosis	2 weeks	Normal	Left	-	-	Superomedial

10	17 years	Male	Proptosis, blurred vision	12 weeks	Normal	Right	-	5	Superomedial

11	10 months	Female	Proptosis, orbital mass	8 weeks	Normal	Both	-	-	Superior

12	9 years	Male	Proptosis, diminution of vision	8 weeks	Disc edema	Right	-	11	Superotemporal

SMN = submandibular lymph nodes; PAN = preauricular lymph nodes; ACN = anterior cervical lymph nodes.

Ocular features were bilateral in 4 patients, right eye was involved in 4 and the left eye involved in 4 patients respectively. Lymphadenopathy was present in 5 patients, the submandibular lymph nodes being the most commonly involved, (Table 1). An orbital mass was palpable in 11 patients, commonly located superior or superotemporally.

A diagnosis of acute myeloid leukemia was established by incision biopsy in 4 patients, 2 of whom further underwent peripheral blood smear testing and bone marrow biopsy which revealed the presence of systemic involvement. Imprint smears of the fresh tissues performed in 2 cases showed the presence of large blasts with scant cytoplasm and a large nucleus with 2–3 nucleoli and fine chromatin pattern. The smears were useful in confirming a hematopoeitic malignancy and in ruling out other tumors. In 8 patients diagnosis was established by peripheral blood smear examination alone which revealed a diagnosis of acute myeloid leukemia, (Table 2). Presence of more than 30% blasts in the peripheral smear was considered diagnostic of acute myeloid leukemia. Blasts were seen as large cells (2–3 times the size of a mature lymphocyte, with a high nuclear/cytoplasmic ratio, a round to indented nucleus with fine nuclear chromatin and 1–3 nucleoli. Myeloperoxidase positivity was noted in all 4 incision biopsy specimens. Once the diagnosis was established all the patients were referred to a pediatric oncologist for further management and were not followed up by us.

**Table 2:** Diagnostic tests

No	Incision biopsy	Peripheral blood smear	Bone marrow	Myeloperoxidase
1	-	+	Positive	-

2	+	-	-	+

3	-	+	-	-

4	-	+	-	-

5	+	+	Positive	+

6	-	+	-	-

7	+	+	Positive	+

8	+	-	-	+

9	-	+	-	-

10	-	+	-	-

11	-	+	Positive	-

12	-	+	Positive	-

## Discussion

Granulocytic sarcoma is a rare presentation of acute myeloid leukemia. Even though it can present from infancy to old age, it most commonly affects children and young adults. In our series the median age at presentation was 6 years. Zimmerman reported a median age of 7 years (range 1–61 years) and Stockl et al 8.8 years in their studies [2,6]. Though previous studies have noted a slight male preponderance with a ratio of about 1.5:1, we had more females affected than males [5,6]. The incidence of this tumor is more in African, Asian, Latin American and Middle Eastern children [2]. Ghose et al in their study of 86 Indian children with AML, reported orbital masses in 9.3% cases [7]. In a study of Turkish children with acute myelomonocytic leukemia, granulocytic sarcoma was reported to be present in 20 of the 56 (36%) children [8]. Templeton in his study of orbital tumors in Africa noted that the second most common orbital malignancy in children after Burkitt's lymphoma was granulocytic sarcoma [9].

Granulocytic sarcoma is thought to originate in the bone marrow and the cells are believed to spread via the Haversian canals to collect in the subperiosteum and form a soft tissue mass [1]. They more commonly affect the skeletal system, commonly the ligaments or periosteum. In cases with head and neck involvement they commonly affect the orbit or epidural space [10]. These tumors most commonly affect the skull, orbit, paranasal sinuses, spine, ribs, sacrum and sternum, involvement being related to the active hematopoeisis at these sites [1]. It can also involve the lymph nodes, skin and kidney [2]. In the autopsy study from Japan by Liu et al, bone was the most common site of involvement [11].

In our series the most common presenting features were an orbital mass and proptosis. Various other studies have described proptosis as the most common presenting feature [9-11], (Table 3). Zimmerman and Font noted that 88% of their patients presented with proptosis [6]. Sudden onset of bilateral proptosis with extraocular muscle infiltration has also been reported [12]. We also noted the presence of retinal and preretinal hemorrhages. The presence of the characteristic pale centered hemorrhages should be viewed with a high index of suspicion of acute myeloid leukemia. Granulocytic sarcoma can also present as ptosis, lacrimal gland involvement, conjunctival mass, iridic and diffuse uveal involvement [2,5].

**Table 3:** Summary of major case series of orbital leukemic tumors

Study	Number of cases	Age(years)	Male/Female ratio	Orbital disease on initial presentation
Stockl et al^2^	7	8.8	2.5:1	4/7

Zimmerman and Font^6^	33	7	1.5:1	29/33

Cavdar et al^8^	33	7.3	4.5:1	32/33

Bidar et al^13^	27	8	2.4:1	4/27

Current study	12	8.8	0.7:1	12/12

The onset of orbital granulocytic sarcoma in relation to systemic AML can be variable. The orbit can be involved even before the bone marrow and peripheral blood show features of the malignancy. In Zimmerman's series, in 88% of the cases the orbital granulocytic sarcoma developed before the development of systemic leukemia [6]. Various reports have described the occurrence of orbital GS before the development of systemic leukemia [2,6,8]. Systemic features usually develop within a year in these patients. In Zimmerman's series, 12 of the 29 developed signs of leukemia within 2 months[6]. Stockl et al in their series reported that 2 of their 7 patients had orbital lesions and the systemic disease simultaneously[2]. In our case series all the patients were noted to have orbital disease and systemic involvement concurrently. Cavdar et al in their series from Turkey reported that 19 of the 20 children had abnormal blood counts at initial presentation[8]. Cavdar et al biopsied only 12 of his 33 patients and Bidar et al biopsied only 2 of 27 patients, both of whom had presented with orbital disease prior to diagnosis of systemic malignancy[13]. We had 4 patients who underwent an incision biopsy at the first instance and this led to the diagnosis of systemic leukemia. These were patients who were seen before the year 2000. In 8 patients we established the diagnosis solely based on the peripheral smear, as after the year 2000, we subjected all children presenting with proptosis to a peripheral blood smear as part of the routine workup. If the diagnosis can be established on a non invasive test like a peripheral blood smear, one can avoid surgical intervention. In our series the median duration of symptoms was 4 weeks. In Cavdar's series the median duration of orbital symptoms was 8 weeks before the diagnosis of leukaemia[8].

The diagnosis of this tumor can be challenging especially when there are no signs of systemic leukemia. In the presence of systemic malignancy, a peripheral blood smear or a bone marrow biopsy may provide useful clues to the diagnosis. Zimmerman et al reported that the most common misdiagnosis was malignant lymphoma[6]. However orbital lymphomas are more common in adults and any such diagnosis in a child should be reinvestigated to rule out granulocytic sarcoma[5]. This tumor can also be confused with rhabdomyosarcomas, neuroblastomas and Burkitt's lymphoma. Neuroblastomas and Ewing's sarcoma usually cause more bony destruction[1,7]. Clinical features are not specific to aid in the diagnosis. However one constant feature we found was the superior or superotemporal location of the tumor in 11 of 12 patients. Radiological features are again not characteristic. The computed tomography (CT) findings have been described as a focal homogenously enhancing lesion with well-defined margins, usually isodense to muscle [1]. The tumors can mould to the surrounding structures. It is usually confined to the orbit and spread to the paranasal sinuses is rare. On T2-weighted MRI it is isointense to white matter, muscle and bone marrow, while on T1-weighted MRI it is slightly hyperintense[1,5]. The tumor shows a marked contrast enhancement to gadopentetate dimeglumine[3,11].

Evaluation of the peripheral smear is an invaluable tool in the diagnosis if the systemic manifestations are already present as in our series. Previous reports have highlighted the role of this inexpensive investigation in all cases of childhood proptosis[7,12]. The peripheral smear reveals the presence of immature blast cells. The total leucocyte count is usually high with a relative neutropenia. Bone marrow examination and flow cytometry should be routinely performed to confirm the diagnosis. Squash and imprint cytology could aid in diagnosis of this hematologic malignancy [13]. Though a definite distinction cannot be made from large cell lymphoma, the presence of singly scattered blasts, with relatively abundant pale staining cytoplasm, 2–3 nucleoli, cytoplasmic granules and Auer rods could help in suggesting extramedullary leukemic deposits [14]. Romanowsky stained (Giemsa) air-dried smears are useful in interpreting the hematologic lesions of leukemia and lymphoma. Immunocytochemistry or immunohistochemistry using antibody against myeloperoxidase could confirm the diagnosis. Hematoxylin eosin stain reveals the presence of cells of myeloid lineage with eosinophilic cytoplasmic granules. Auer rods may be absent in routine Romanowsky stained specimens. Mc Carty et al have reported that sudanophilic (Sudan black B) and myeloperoxidase staining can demonstrate fusiform and spindle shaped particles, phi bodies and rods on light microscopy in these patients [15]. The diagnosis is also dependant on the amount of granulocytic differentiation. Niemen et al noted that poorly differentiated tumors are diagnosed correctly 47% of the time and well differentiated tumors 54% of the time[16]. The Leder stain (naphtol-AS-O-chlororacetate esterase) measures the esterase activity and can be performed on paraffin stained slides. Diagnosis can be established in nearly 75% of the cases using this stain[2]. Immunohistochemical stains using antilysozyme antibody has been reported to be positive in upto 90% of the cases. Stockl et al reported MAC387 positivity was present in all their seven cases of granulocytic sarcoma[2].

The prognosis for patients with granulocytic sarcoma depends on the course of the underlying systemic malignancy[1,5]. Initiating treatment early can improve the prognosis and outcome. Chemotherapy is the mainstay of treatment. The presence of orbital granulocytic sarcoma does not significantly alter the survival in patients with AML[5]. The rate of remission following chemotherapy also does not seem to be significantly affected due to the presence of granulocytic sarcoma.

## Conclusion

Granulocytic sarcoma is a rare cause of childhood proptosis. A peripheral blood smear should be performed in all cases of sudden onset proptosis or an orbital mass in children and young adults. It should be complemented with a bone marrow biopsy especially in cases of aleukemic leukemia and biopsy of the orbital lesion especially in cases where the blood smear is inconclusive. The use of special stains and immunohistochemical techniques can help further in establishing the diagnosis in orbital GS. Early treatment and diagnosis is the key to prolonging the survival in this aggressive malignancy.

## Methods

**Figure 1:**
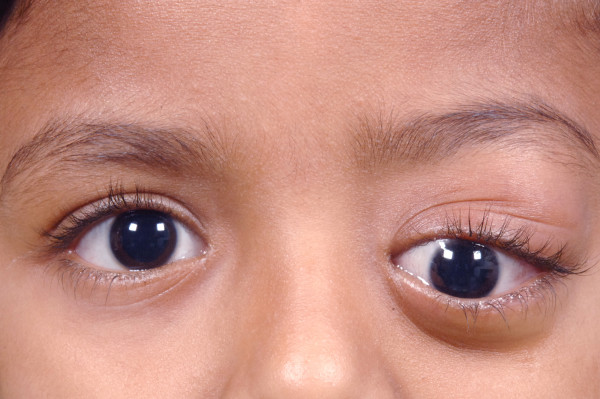
**A 6 year old girl presented with left eye proptosis and downward displacement of the globe**. A firm mass was palpable in the superotemporal quadrant of the left orbit.

**Figure 2:**
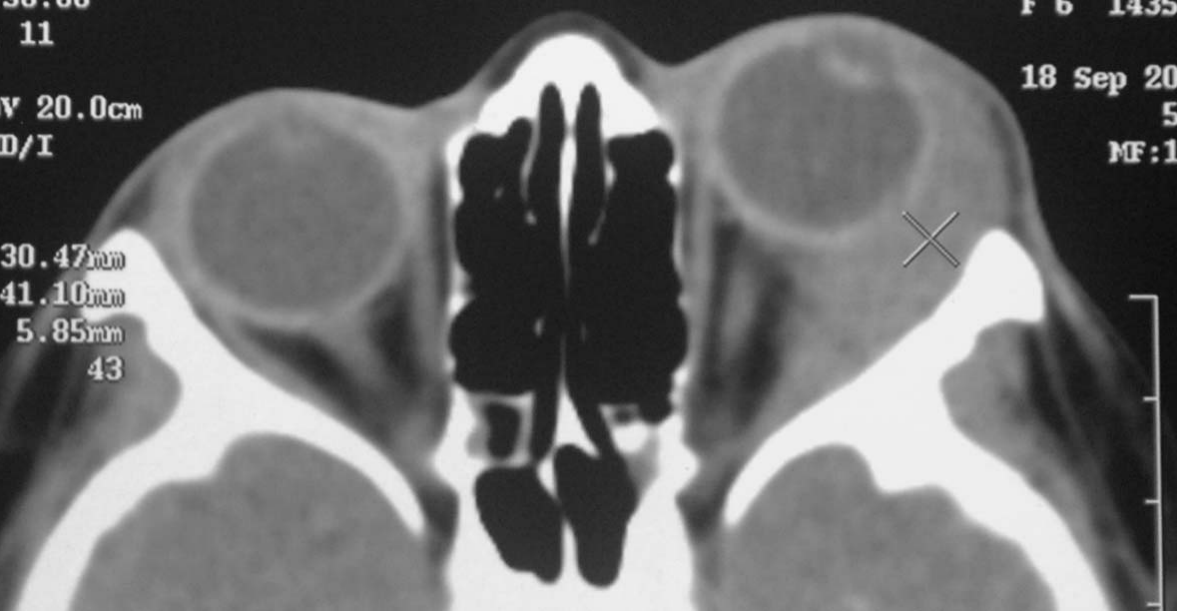
**CT scan axial view revealed a diffuse soft tissue mass involving the left superotemporal orbit**.

**Figure 3:**
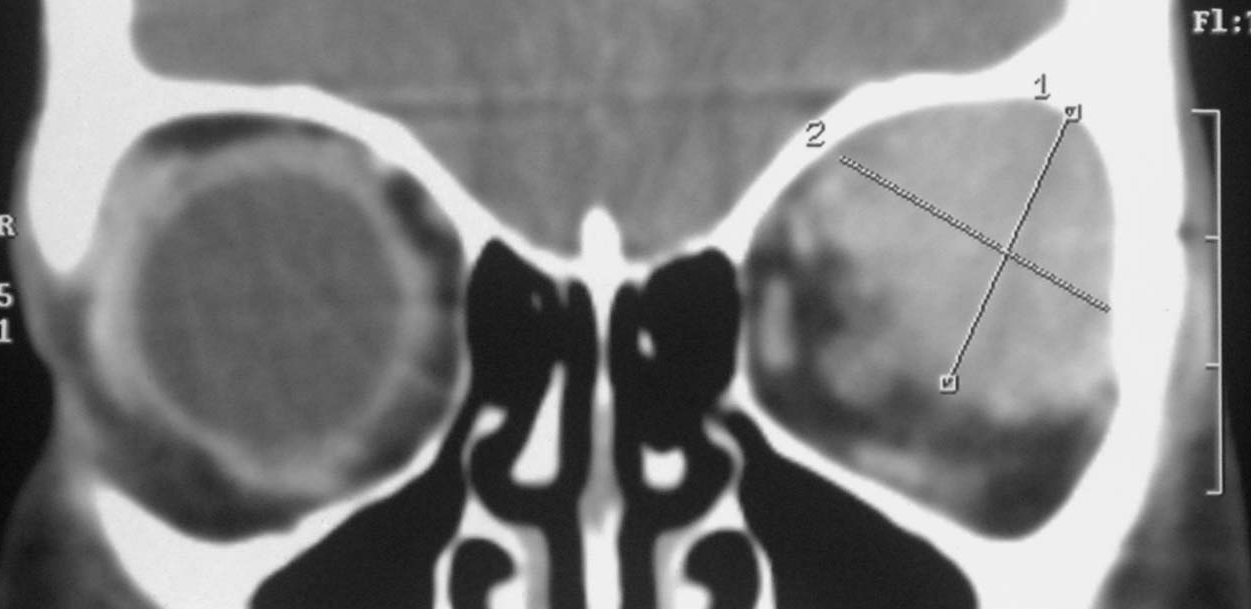
**CT scan coronal view showed the superior and lateral rectus muscles could not be differentiated from the mass**.

**Figure 4:**
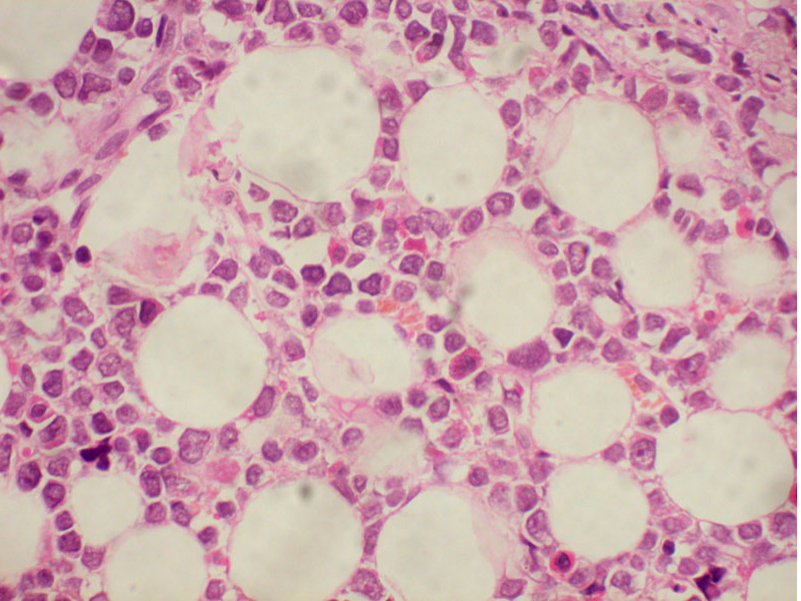
**Histopathology of orbital mass showing a round cell tumor infiltrating the orbital fat tissues (hematoxylin & eosin, × 400)**.

**Figure 5:**
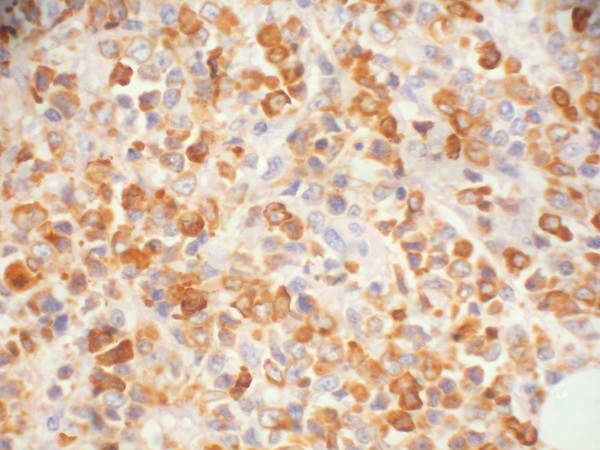
**The tumor cells are strongly immunoreactive to myeloperoxidase (DAB, × 400)**.

**Figure 6:**
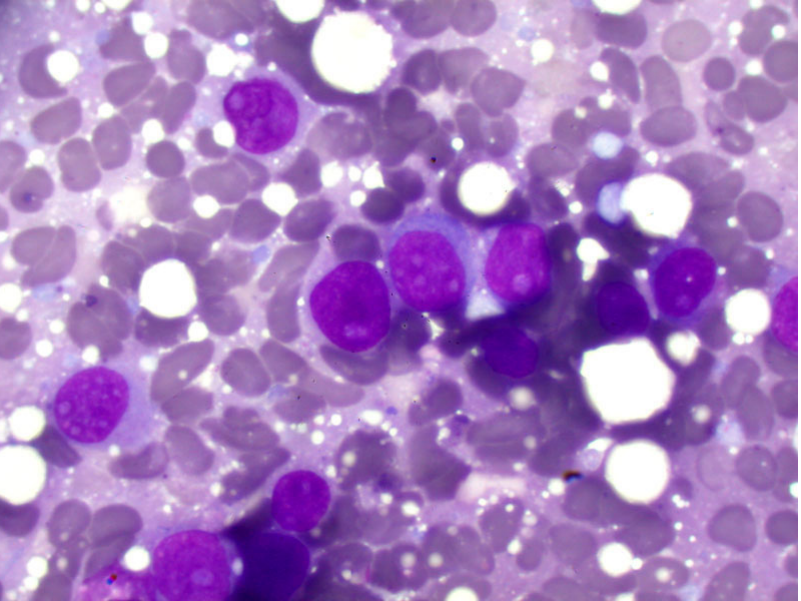
**The imprint smears of the biopsy show clumps of large round cells appearing like blasts (Giemsa, × 400)**.

**Figure 7:**
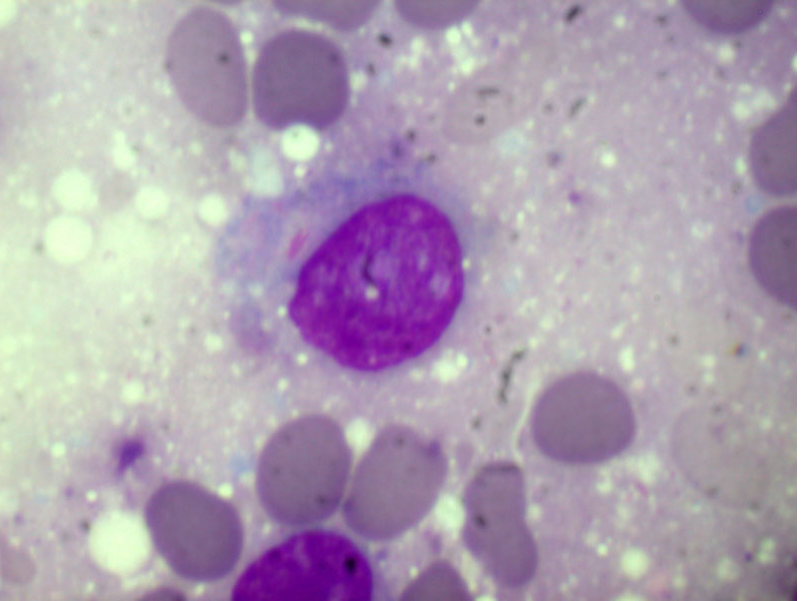
**Higher magnification of the same shows the presence of Auer rod in the cytoplasm of cells (Giemsa, × 1000)**.

A retrospective review of the records of all the patients in the ophthalmic pathology service was performed from January 1998 to September 2008. There were 13 cases of leukemia involving the eye or the orbit (for examples and further detail, see figures 1, 2, 3, 4, 5, 6, 7). One of them had only intraocular manifestations and was excluded from this study. Twelve cases with orbital manifestations were included in the case series.

Medical records of these patients were reviewed for the demographic details, clinical presenting features and radiologic findings. Diagnosis was established by peripheral blood smear or incision biopsy and in addition some cases underwent bone marrow biopsy. The peripheral blood smear examination and bone marrow cytology was done by an experienced pathologist (GKV) as per the FAB classification. In cases that underwent incisional biopsy, intraoperative diagnosis was attempted by squash and imprint cytology. Myeloperoxidase staining was performed on the smears or biopsy specimens. All the patients were referred to a pediatric oncologist for further management.

## Consent

Written informed consent was obtained from the patient for publication of this case report and accompanying images. A copy of the written consent is available for review by the Editor-in-chief of this journal.

## Competing interests

The authors declare that they have no competing interests.

## Authors' contributions

RM conceived the idea, collected data and drafted the manuscript; GKV performed histopathologic studies and contributed to the manuscript writing; SGH participated in the study design and provided critical inputs; MN and VR participated in the study design and provided critical inputs. All authors read and approved the final manuscript.

## References

[B1] Stein-Wexler R, Wootton-Gorges SL, West DC (2003). Orbital granulocytic sarcoma: an unusual presentation of acute myelocytic leukemia. Pediatr Radiol.

[B2] Stockl FA, Dolmetsch AM, Saornil MA (1997). Orbital granulocytic sarcoma. Br J Ophthalmol.

[B3] Uyesugi WY, Watabe J, Petermann G (2000). Orbital and facial granulocytic sarcoma (chloroma): a case report. Pediatr Radiol.

[B4] Rappaport H (1966). Tumors of the hematopoeitic system. Atlas of Tumor Pathology.

[B5] Davis JL, Park DW, Font RL (1985). Granulocytic sarcoma of the orbit. A histopathologic study. Ophthalmology.

[B6] Zimmerman LE, Font RL (1975). Ophthalmic manifestations of granulocytic sarcoma (myeloid sarcoma or chloroma). The Third Pan American Association of Ophthalmology and Journal of Ophthalmology Lecture. Am J Ophthalmol.

[B7] Sethi A, Ghose S, Gujral S, Jain P, Kumar R (2001). Childhood proptosis: the invaluable but overlooked peripheral blood smear. Indian J Ophthalmol.

[B8] Cavdar AO, Babacan E, Gozdasoglu S (1978). Ocular granulocytic sarcoma (chloroma) with acute myelomonocytic leukemia in Turkish children. Cancer.

[B9] Templeton AC (1971). Orbital tumours in African children. Br J Ophthalmol.

[B10] Porto L, Kieslich M, Schwabe B, Zanella FE, Lanfermann H (2004). Granulocytic sarcoma in children. Neuroradiology.

[B11] Liu PI, Ishimaru T, Mc Gregor DH, Akada H, Steer A (1973). Autopsy study of granulocytic sarcoma (chloroma) in patients with myelogenous leukemia, Hiroshima- Nagasaki 1949–1969. Cancer.

[B12] Panda A, Dayal Y (1984). Acute proptosis in myeloid leukaemia. Indian J Ophthalmol.

[B13] Vemuganti GK, Naik M, Honavar SG, Shekar GC (2004). Rapid Intraoperative Diagnosis of Tumors of the Eye and Orbit by Squash and Imprint Cytology. Ophthalmology.

[B14] Bidar M, Wilson MW, Laquis SJ (2007). Clincal and imaging characteristics of orbital leukemic tumors. Ophthal Plast Reconstr Surg.

[B15] Mc Carty KS, Wortmen J, Daly J, Rundles RW, Hanker JS (1980). Chloroma without evidence of leukemia: facilitated light microscopic diagnosis. Blood.

[B16] Neiman RS, Barcos M, Berard C (1981). Granulocytic sarcoma: a clinicopathologic study of 61 biopsied cases. Cancer.

